# Post-discharge spirometry evaluation in patients recovering from moderate-to-critical COVID-19: a cross-sectional study

**DOI:** 10.1038/s41598-024-67536-2

**Published:** 2024-07-16

**Authors:** Chee-Shee Chai, Muhammad Amin Bin Ibrahim, Nur Amira Binti Azhar, Zulaika Binti Roslan, Rozila Binti Harun, Swarna Lata Krishnabahawan, Aruna A. P. Karthigayan, Roqiah Fatmawati Binti Abdul Kadir, Busra Binti Johari, Diana-Leh-Ching Ng, Benedict-Lim-Heng Sim, Chong-Kin Liam, Abdul Razak Bin Muttalif, Ahmad Hata Bin Rasit, Kalaiarasu M. Peariasamy, Mohammed Fauzi Bin Abdul Rani

**Affiliations:** 1 Department of Medicine, Faculty of Medicine and Health Science, University Malaysia Sarawak, Kota Samarahan, Sarawak Malaysia; 2 Department of Internal Medicine, Faculty of Medicine, University Technology MARA, Sungai Buloh, Selangor Malaysia; 3 Clinical Research Centre, Sungai Buloh Hospital, Ministry of Health Malaysia, Sungai Buloh, Selangor Malaysia; 4 Department of Medicine, Sungai Buloh Hospital, Ministry of Health Malaysia, Sungai Buloh, Selangor Malaysia; 5 Department of Radiology, Faculty of Medicine, University Technology MARA, Sungai Buloh, Selangor Malaysia; 6 Department of Medicine, Faculty of Medicine, University of Malaya, Kuala Lumpur, Malaysia; 7 Department of Medicine, Faculty of Medicine, MAHSA University Malaysia, Jenjarom, Selangor Malaysia; 8 Department of Orthopaedics, Faculty of Medicine and Health Science, University Malaysia Sarawak, Kota Samarahan, Sarawak Malaysia; 9 Institute for Clinical Research, National Institutes of Health, Ministry of Health Malaysia, Shah Alam, Selangor Malaysia

**Keywords:** COVID-19, Spirometry, Restrictive, Obstructive, PRISm, Radio-imaging, Diseases, Risk factors

## Abstract

Understanding the prevalence of abnormal lung function and its associated factors among patients recovering from COVID-19 is crucial for enhancing post-COVID care strategies. This study primarily aimed to determine the prevalence and types of spirometry abnormalities among post-COVID-19 patients in Malaysia, with a secondary objective of identifying its associated factors. Conducted at the COVID-19 Research Clinic, Faculty of Medicine, University Technology MARA, from March 2021 to December 2022, this study included patients at least three months post-discharge from hospitals following moderate-to-critical COVID-19. Of 408 patients studied, abnormal spirometry was found in 46.8%, with 28.4% exhibiting a restrictive pattern, 17.4% showing preserved ratio impaired spirometry (PRISm), and 1.0% displaying an obstructive pattern. Factors independently associated with abnormal spirometry included consolidation on chest X-ray (OR 8.1, 95% CI 1.75–37.42, *p* = 0.008), underlying cardiovascular disease (OR 3.5, 95% CI 1.19–10.47, *p* = 0.023), ground-glass opacity on chest X-ray (OR 2.6, 95% CI 1.52–4.30, *p* < 0.001), and oxygen desaturation during the 6-min walk test (OR 1.9, 95% CI 1.20–3.06, *p* = 0.007). This study highlights that patients recovering from moderate-to-critical COVID-19 often exhibit abnormal spirometry, notably a restrictive pattern and PRISm. Routine spirometry screening for high-risk patients is recommended.

## Introduction

The Coronavirus 2019 (COVID-19) pandemic is the worst-ever global health emergency, resulting in substantial human casualties and economic downturn. As of 6th March 2024, global COVID-19 infections have reached 704 million, leading to over seven million fatalities^1^. Even though the World Health Organization (WHO) no longer considers COVID-19 a public health emergency of international concern^2^, the continual emergence of new virus variants poses a persistent threat to the end of the pandemic.

Viruses responsible for Severe Acute Respiratory Syndrome (SARS), Middle East Respiratory Syndrome (MERS), and COVID-19 primarily target the lower respiratory tract, leading to acute lung injuries like pneumonia and acute respiratory distress syndrome (ARDS)^3^. Survivors of SARS and MERS demonstrated abnormal lung function, reduced effort tolerance, and impaired quality of life months or even years after the illness^4–6^. Recent studies conducted in China highlighted that abnormal lung function was observed in 47.2% of hospitalized COVID-19 patients upon discharge^7^, 75.4% after a month^8^, and 25.5% after three months^9^. A study of previously hospitalized COVID-19 survivors in China reported dyspnea rates of 26.1% at six months and 14.1% at two years, with impaired functional status observed in 14.1% at six months and 8.4% at two years^10^. Nearly a quarter experienced impaired health-related quality of life (HRQOL) due to somatic symptoms or psychiatric symptoms at six months, with somatic symptoms remaining constant while psychiatric symptoms halved by two years^10^. In various respiratory diseases, lung function, dyspnea symptoms, and functional status are often negatively correlated with HRQOL^11^.

Malaysia has reported 5.27 million COVID-19 cases to date, with a recovery rate of 98.9%^1^. Routine assessment of lung function in patients recovering from COVID-19, however, remains a major challenge here due to a few constraints. First, conducting widespread lung function tests is time-consuming, costly, and manpower intensive. Second, equipment (such as spirometer and body plethysmograph) and expertise required to perform lung function tests are only available in selected tertiary healthcare centres. Third, non-respiratory clinicians often have difficulty interpreting the results of lung function tests. Fourth, the management strategies for abnormal lung function following COVID-19 remain unclear, particularly lacking standardized guidelines. Thus, only a very small proportion of patients recovering from COVID-19 were offered lung function tests.

Research looking into the prevalence of abnormal lung function and its associated factors among patients recovering from COVID-19 in Malaysia is essential to help healthcare authorities develop follow-up strategies to enhance post-COVID care. This study focuses on evaluating lung function in patients with COVID-19 at least three months after their hospital discharge, aiming to determine the prevalence and types of abnormal spirometry results as primary objectives. The secondary objective is to identify the factors associated with spirometry abnormalities.

## Methods

### Study design and patients

This is a cross-sectional study of patients attending the COVID-19 Research Clinic at the Faculty of Medicine, University Technology MARA (UITM) in Malaysia, from March 2021 to December 2022. The inclusion criteria were Malaysians aged eighteen years and above, with confirmed COVID-19 via validated reverse transcription-polymerase chain reaction method, who had moderate-to-critical illness according to the WHO classification^12^, and were at least three months post-discharge from either the Sungai Buloh Hospital or the UITM Medical Centre^13^. Patients with pre-existing chronic lung diseases before COVID-19, including bronchial asthma, as well as individuals who were pregnant, completely immobilized, had uncontrolled psychiatric illness, or were contraindicated for spirometry were excluded. The exclusion criteria were determined based on patients’ electronic records or interviews.

A minimum sample size of 386 subjects was determined using the formula for a cross-sectional study—sample size = Z_1-α_^2^*p*(1—p)/d^2^ ^14^. Z represented the confidence interval at 95%, d denoted the margin of error at 5%, and p referred to the proportion of abnormal lung function (52.7%) among SARS survivors in a previous study^5^. All patients provided written informed consent before participating in the study. The study received ethics approval from the Medical Research Ethics Committee of the Ministry of Health Malaysia **(**NMRR-20-2011-56330 (IIR) and the respective hospitals, and it was conducted in adherence to the Declaration of Helsinki.

### Procedure and outcomes

Eligible patients were consecutively identified from the COVID-19 registry of Sungai Buloh Hospital and UITM Medical Centre. Those meeting all inclusion criteria and having none of the exclusion criteria were scheduled for early physical appointments at the COVID-19 Research Clinic.

1. Demographic, clinical, and hospitalization data:

Demographic, clinical, and hospitalization data were gathered through face-to-face interviews and the electronic records. Demographic information included age, gender, and ethnicity, while clinical details included smoking status and the presence of underlying chronic diseases. Hospitalization data included the duration of illness before admission, length of hospital stays, COVID-19 severity at presentation, the most severe COVID-19 episode during hospitalization, pharmacotherapy administered, respiratory support provided, the occurrence of respiratory complications, and details regarding intensive care unit (ICU) admission, including its length of stay.

The severity of COVID-19 was defined according to the WHO classification as: asymptomatic, mild (symptomatic without pneumonia), moderate (pneumonia without hypoxia), severe (pneumonia with hypoxia requiring oxygen supplementation), and critical (critically ill, such as ARDS, sepsis, or septic shock)^12^. Available treatments for COVID-19 during the study period included corticosteroids, hydroxychloroquine, immunomodulators (tocilizumab, interferon beta, and interferon alpha), and antivirals (favipiravir, lopinavir-ritonavir, ritonavir, and atazanavir)^15^. Respiratory support was categorized into oxygen supplementation by nasal cannulae, venti-mask, or high-flow mask, non-invasive mechanical ventilation (NIV) or nasal high flow (NHF), and invasive mechanical ventilation (IMV)^16,17^. Common respiratory complications of COVID-19 that were recorded included ARDS, pulmonary embolism, pneumothorax, and pleural effusion^18,19^.

2. Patients reported outcomes (PROs):

Patients were instructed to independently complete the modified Medical Research Council (mMRC) dyspnea scale and the post-COVID-19 Functional Status (PCFS) scale with minimal assistance from investigators. The mMRC and PCFS were interpreted as per the original validation of the questionnaire^20,21^. A higher score indicates a greater degree of symptom severity and impairment, respectively.

3. Lung function tests:

Spirometry was conducted using SpiroUSB™ (Vyaire Medical, Chicago, IL) to obtain dynamic lung volumes, including the forced expiratory volume in one second (FEV_1_) and forced vital capacity (FVC). The cut-off value of ≥ 80% of the predicted was deemed normal for both parameters. Spirometry results were categorized into four groups: normal spirometry—normal FEV_1_, normal FVC, and FEV_1_/FVC > 0.7; restrictive pattern—reduced or normal FEV_1_, reduced FVC, and FEV_1_/FVC > 0.7; obstructive pattern—reduced FEV_1_, reduced or normal FVC, and FEV_1_/FVC < 0.7; and preserved ratio impaired spirometry (PRISm)—reduced FEV_1_, normal FVC¸ and FEV_1_/FVC > 0.7^22,23^. For patients with an obstructive pattern, post-bronchodilator spirometry was performed to identify reversible airflow obstruction. Those with a restrictive pattern were scheduled for static lung volumes and diffusion capacity measurement within two weeks using PFT Vyntus Bodybox™ (Vyaire Medical, Chicago, IL). The parameters measured included residual volume (RV), total lung capacity (TLC), diffusion capacity for carbon monoxide (DLCO), and carbon monoxide transfer coefficient (DLCO/Va). All lung function tests were conducted by certified respiratory technicians following the American Thoracic Society (ATS) and European Respiratory Society guidelines^24,25^.

4. Cardiopulmonary functional tests:

Patients underwent a 6-min walk test (6MWT) under the guidance of a certified respiratory physiotherapist, following the ATS guideline^26^. Their pulses and oxygen saturation were continuously monitored using the Nonin® WristOx2 ™ 3150 Bluetooth Pulse Oximeter. A 1-min sit-to-stand test (1MSTS) guided by the same respiratory physiotherapist followed and in accordance with the procedure outlined in a previous study^27^. Both assessments utilized a digital stopwatch for time measurement, and the Borg scale was employed to assess the severity of dyspnea and fatigue. Both tests were sensitive for respiratory diseases but not specific for abnormal spirometry^26,27^.

5. Radio-imaging:

All patients underwent a standard posterior-anterior chest X-ray examination. Only those demonstrating a restrictive pattern in spirometry were scheduled for high-resolution computed tomography (HRCT) of the lungs within one month. Two radiologists, blinded to patients’ information, independently reviewed the chest X-ray images to identify consolidation, ground-glass opacity (GGO), and lung parenchymal reticulation which were commonly reported in previous COVID-19 literature^28–31^. On a chest X-ray, consolidation was defined as a homogeneous opacification that obscures airway walls and blood vessels; GGO was defined as a hazy lung radiopacity with indistinct pulmonary vessel edges; and reticulation was defined as a collection of numerous small linear opacities, according to the Fleischner Society Glossary^32^. HRCT images, when available, were also reviewed to detect these findings, as well as organizing pneumonia (OP) and other relevant abnormalities such as lung nodules, atelectasis, pleural effusion or thickening, diaphragmatic elevation, cardiomegaly, and fractures, if present. To prevent cross-referencing, all chest X-ray images were reported before any HRCT evaluations were conducted. The two radiologists did not refer to the chest X-ray results when reporting the HRCT images, and vice versa. The reporting of HRCT scans was done randomly, so the radiologists who reported the HRCT might not be the same one who evaluated the chest X-ray. Lung involvement severity was assessed using the CT-score method developed by Kunhua Li et al^33^. Each lobe received a score ranging from 0 to 5 based on its level of involvement: 0 (0%), 1 (< 5%), 2 (5–25%), 3 (26–49%), 4 (50–75%), and 5 (> 75%). The total score, representing cumulative involvement across all lobes, ranged from 0 to 25 points.

### Statistical analyses

Categorical variables are presented as percentages, while continuous variables are presented as mean ± standard deviation (SD). Patients were categorized into those with normal versus those with abnormal spirometry for two-group comparisons, as well as normal versus restrictive pattern or PRISm/obstructive pattern spirometry for three-group comparisons. Between-group differences were assessed using an independent *t*-test for continuous variables and a Chi-Square test for categorical variables. A two-sided *p*-value of less than 0.05 was considered statistically significant.

For multivariate analyses, variables exhibiting significant two-sided *p*-values in the univariate analyses were included as covariates in binary logistic regression and multinomial logistic regression. The latter analysis excluded variables showing multicollinearity (variance inflation factor > 5). The analysis aimed to derive odds ratios (OR), 95% confidence intervals (95% CI), and two-sided *p*-values. Statistical analysis was conducted using the Statistical Package for the Social Sciences (SPSS for Windows version 25.0, SPSS Inc, Chicago, IL, USA).

## Results

### Sociodemographic and clinical characteristics

**Figure 1:**
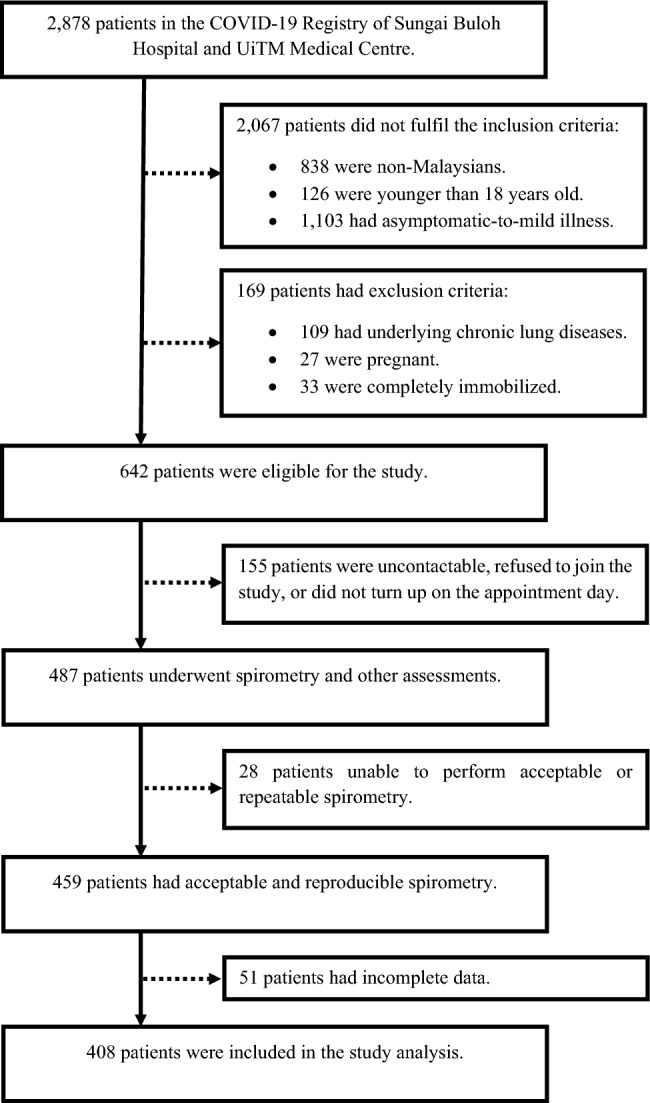
Algorithm of patients’ recruitment into the study.

A total of 408 patients were included in the study (Fig. 1). The sociodemographic and clinical characteristics of these patients are presented in Table 1. The mean age of the patients was 51.6 ± 13.32 years. The majority were male (59.8%), of Malay ethnicity (71.8%), and had underlying chronic diseases (63.7%). The most common disease was hypertension (43.4%), followed by diabetes mellitus (30.6%), obesity (17.9%), cardiovascular disease (4.9%), chronic kidney disease (1.5%), chronic liver disease (0.5%), and cerebrovascular disease (0.2%). Only 23.3% of the patients were current or ex-smokers.

**Table 1:** Demographic and clinical data of the patients.

Parameters	All patients, n = 408	Result of Spirometry				*p*-value*	*p*-value^#^
		Normal, n = 217	Abnormal, n = 191	Restrictive, n = 116	PRISm and obstructive, n = 75		
Age, Mean (± SD), years	51.6 ± 13.32	48.8 ± 13.48	54.9 ± 12.39	54.0 ± 12.45	56.3 ± 12.24	< 0.001	< 0.001
Gender, n (%)							
Male	244 (59.8)	127 (58.5)	117 (61.3)	59 (50.9)	58 (77.3)	0.574	0.001
Female	164 (40.2)	90 (41.5)	74 38.7)	57 (49.1)	17 (22.7)		
Ethnicity, n (%)							
Malay	293 (71.8)	155 (71.4)	138 (72.3)	82 (70.7)	56 (74.7)	0.091	0.245
Chinese	90 (22.1)	54 (24.9)	36 (18.8)	22 (19.0)	14 (18.7)		
Indian	23 (5.6)	7 (3.2)	16 (8.4)	11 (9.5)	5 (6.7)		
Others	2 (0.5)	1 (0.5)	1 (0.5)	1 (0.9)	0		
Smoking status, n (%)							
Never smoker	313 (76.7)	172 (79.3)	141 (73.8)	89 (76.7)	52 (69.3)	0.194	0.215
Current and ex-smoker	95 (23.3)	45 (20.7)	50 (26.2)	27 (23.3)	23 (30.7)		
Underlying chronic diseases, n (%)							
No	148 (36.3)	91 (41.9)	57 (29.8)	28 (24.1)	29 (38.7)	0.011	0.005
Yes	260 (63.7)	126 (58.1)	134 (70.2)	88 (75.9)	46 (61.3)		
Obesity	*73 (17.9)*	*42 (19.4)*	*31 (16.2)*	*21 (18.1)*	*10 (13.3)*	*0.411*	*0.501*
Diabetes mellitus	*125 (30.6)*	*59 (27.2)*	*66 (34.6)*	*47 (40.5)*	*19 (25.3)*	0.107	0.023
Hypertension	*177 (43.4)*	*75 (34.6)*	*102 (53.4)*	*68 (58.6)*	*34 (45.3)*	< 0.001	< 0.001
Cardiovascular disease	*20 (4.9)*	*5 (2.3)*	*15 (7.9)*	*9 (7.8)*	*6 (8.0)*	0.01	0.035
Cerebrovascular disease	*1 (0.2)*	*1 (0.5)*	*0*	*0*	*0*	0.348	0.643
Chronic liver disease	*2 (0.5)*	*0*	*2 (1.0)*	*2 (1.7)*	*0*	0.131	0.08
Chronic kidney disease	*6 (1.5)*	*2 (0.9)*	*4 (2.1)*	*2 (1.7)*	*2 (2.7)*	0.326	0.537
Others	*39 (9.6)*	*17 (7.8)*	*22 (11.5)*	*12 (10.3)*	*10 (13.3)*	0.207	0.356

In italic: only the number of patients with the respective underlying chronic diseases is presented; *, the *p*-value for differences between patients with normal and abnormal spirometry; #, the *p*-value for differences between patients with normal, restrictive pattern, and PRISm and obstructive pattern spirometry.

### Hospitalization and management of the patients

The patients were admitted to the hospital after a mean duration of 8.7 ± 5.32 days from symptom onset and the mean hospitalization duration was 13.0 ± 10.62 days. Most of the patients had severe illness (61.3%) on admission (Table 2). Subsequently, 28.9%, 55.6%, and 15.5% developed critical, severe, and moderate illness during their hospital stay, respectively. Corticosteroids (83.1%) were the most frequently administered medication, followed by antivirals (37.0%), immunomodulators (14.2%), and hydroxychloroquine (13.7%). Among patients requiring respiratory support (78.4%), 47.8% received supplemental oxygen only, 13.2% had NIV or HFNO, and 17.4% underwent IMV. Respiratory complications occurred in 26.5% of patients, with pulmonary embolisms accounting for 24.3% of these cases. For the 43.4% of patients who were admitted to the ICU, the mean duration of ICU stay was 10.6 ± 16.32 days.

**Table 2:** Hospitalization data of the patients.

Parameters	All patients, n = 408	Result of Spirometry				*p*-value*	*p*-value^#^
		Normal, n = 217	Abnormal, n = 191	Restrictive, n = 116	PRISm and obstructive, n = 75		
Duration of illness, Mean (± SD), days	8.7 ± 5.32	8.5 ± 5.07	8.9 ± 5.59	8.7 ± 5.99	9.1 ± 4.93	0.495	0.703
Length of hospital stay, Mean (± SD), days	13.0 ± 10.62	12.1 ± 11.30	14.1 ± 9.70	16.1 ± 10.92	11.1 ± 6.37	0.054	0.001
COVID-19 severity at presentation, n (%)							
Asymptomatic	24 (5.9)	18 (8.3)	6 (3.1)	2 (1.7)	4 (5.3)	0.185	0.107
Mild	56 (13.7)	32 (14.7)	24 (12.6)	11 (9.5)	13 (17.3)		
Moderate	39 (9.6)	22 (10.1)	17 (8.9)	8 (6.9)	9 (12.0)		
Severe	250 (61.3)	126 (58.1)	124 (64.9)	80 (69.0)	44 (58.7)		
Critical	39 (9.6)	19 (8.8)	20 (10.5)	15 (12.9)	5 (6.7)		
Most severe illness during hospitalization, n (%)							
Moderate	63 (15.5)	38 (17.5)	25 (13.1)	7 (6.0)	18 (24.0)	0.052	< 0.001
Severe	227 (55.6)	127 (58.5)	100 (52.4)	56 (48.3)	44 (58.7)		
Critical	118 (28.9)	52 (24.0)	66 (34.5)	53 (45.7)	13 (17.3)		
Pharmacotherapy, n (%)							
No	26 (6.4)	15 (6.9)	11(5.8)	7(6.0)	4 (5.3)	0.634	0.876
Yes	382 (93.6)	202 (93.1)	180 (94.2)	109 (94.0)	71 (94.7)		
Corticosteroids	*339 (83.1)*	*170 (78.3)*	*169 (88.5)*	*108 (93.1)*	*61 (81.3)*	*0.006*	*0.003*
Hydroxychloroquine	*56 (13.7)*	*36 (16.6)*	*20 (10.5)*	*4 (3.4)*	*16 (21.3)*	*0.073*	< *0.001*
Immunomodulators	*58 (14.2)*	*24 (11.1)*	*34 (17.8)*	*28 (24.1)*	*6 (8.0)*	*0.052*	*0.001*
Antivirals	*151 (37.0)*	*84 (38.7)*	*67 (35.1)*	*39 (33.6)*	*28 (37.3)*	*0.448*	*0.656*
Respiratory support, n (%)							
None	88 (21.6)	54 (24.9)	34 (17.8)	12 (10.3)	22 (29.3)	0.083	0.002
Yes	320 (78.4)	163 (75.1)	157 (82.2)	104 (89.7)	53 (70.7)		
Oxygen supplementation by nasal cannula/venti-mask/high flow mask	*195 (47.8)*	*109 (50.2)*	*86 (45.0)*	*49 (42.2)*	*37 (49.3)*	*0.294*	*0.364*
NIV/NHF	*54 (13.2)*	*24 (11.1)*	*30 (15.7)*	*22 (19.0)*	*8 (10.7)*	*0.167*	*0.098*
IMV	*71 (17.4)*	*30 (13.8)*	*41 (21.5)*	*33 (28.4)*	*8 (10.7)*	*0.042*	*0.001*
Respiratory complications, n (%)							
No	300 (73.5)	169 (77.9)	131 (68.6)	81 (69.8)	50 (66.7)	0.034	0.093
Yes	108 (26.5)	48 (22.1)	60 (31.4)	35 (30.2)	25 (33.3)		
ARDS	*5 (1.2)*	*5 (2.3)*	*0*	*0*	*0*	*0.035*	*0.108*
Pulmonary embolism	*99 (24.3)*	*41 (18.9)*	*58 (30.4)*	*35 (30.2)*	*23 (30.7)*	*0.007*	*0.026*
Pneumothorax	*10 (2.5)*	*6 (2.8)*	*4 (2.1)*	*2 (1.7)*	*2 (2.7)*	*0.662*	*0.835*
Pleural effusion	*3 (0.7)*	*2 (0.9)*	*1 (0.5)*	*1 (0.5)*	*0*	*0.639*	*0.71*
ICU admission, n (%)							
No	231 (56.6)	140 (64.5)	91 (47.6)	45 (38.8)	46 (61.3)	0.001	< 0.001
Yes	177 (43.4)	77 (35.5)	100 (52.4)	71 (61.2)	29 (38.7)		
Length of ICU stay, Mean (± SD), days	10.6 ± 16.32	9.6 ± 12.59	11.3 ± 18.69	11.7 ± 19.62	10.5 ± 16.55	0.522	0.774

In italic: only the number of patients with the respective pharmacotherapy, respiratory support, and respiratory complications are presented; *, *p*-value for differences between patients with normal and abnormal spirometry; #, *p*-value for differences between patients with normal, restrictive pattern, and PRISm and obstructive pattern spirometry.

### PROs, cardiopulmonary functional tests, and chest X-ray findings at follow-up

The patients were assessed at a mean duration of 162.6 ± 113.97 days post-hospital discharge (Table 3). They reported a mean mMRC score of 0.9 ± 0.95 and a mean PCFS score of 0.4 ± 0.74. Of 404 patients who completed the 6MWT, 31.4% experienced oxygen desaturation. Among the 402 patients who completed 1MSTS, 29.1% experienced oxygen desaturation. Chest X-ray revealed abnormalities in 33.6% of patients which included GGO (26.0%), lung parenchymal reticulation (10.1%), and consolidation (4.2%).

**Table 3:** PROs, cardiopulmonary functional tests, and chest X-ray findings of the patients.

Parameters	All patients, n = 408	Result of Spirometry				*p*-value*	*p*-value^#^
		Normal, n = 217	Abnormal, n = 191	Restrictive, n = 116	PRISm and obstructive, n = 75		
Time from discharge to follow-up, Mean (± SD), days	162.6 ± 113.97	179.6 ± 125.11	143.5 ± 96.73	121.9 ± 61.46	176.1 ± 127.50	0.001	< 0.001
mMRC score, Mean (± SD)	0.9 ± 0.95	0.8 ± 0.90	1.0 ± 1.0	1.2 ± 1.06	0.6 ± 0.82	0.212	< 0.001
PCFS score, Mean (± SD)	0.4 ± 0.74	0.4 ± 0.74	0.4 ± 0.75	0.5 ± 0.77	0.3 ± 0.69	0.698	0.307
6MWT^							
6MWT distance, Mean (± SD), meter	389.9 ± 78.18	396.2 ± 71.70	382.5 ± 84.69	366.8 ± 73.10	407.0 ± 95.60	0.078	0.001
Oxygen desaturation, n (%)							
No	277 (68.6)	163 (75.1)	114 (61.0)	69 (60.5)	45 (61.6)	0.002	0.009
Yes	127 (31.4)	54 (24.9)	73 (39.0)	45 (39.5)	28 (38.4)		
Baseline oxygen saturation, Mean (± SD)	96.6 ± 1.76	96.8 ± 1.70	96.5 ± 1.81	96.2 ± 1.80	96.9 ± 1.75	0.087	0.006
% Nadir oxygen saturation, Mean (± SD),	93.8 ± 2.95	94.3 ± 2.54	93.2 ± 3.28	92.7 ± 3.26	94.0 ± 3.18	< 0.001	< 0.001
%Oxygen saturation on recovery, Mean (± SD), %	96.5 ± 1.95	96.9 ± 1.56	96.0 ± 2.23	95.7 ± 2.38	96.5 ± 1.90	< 0.001	< 0.001
1MSTS^+^							
Repetitions, n Mean (± SD)	20.6 ± 5.44	20.9 ± 5.17	20.2 ± 5.73	19.6 ± 6.10	21.2 ± 5.00	0.204	0.063
Oxygen desaturation, n (%)							
No	285 (70.9)	166 (77.2)	119 (63.6)	70 (61.4)	49 (67.1)	0.003	0.008
Yes	117 (29.1)	49 (22.8)	68 (36.4)	44 (38.6)	24 (32.9)		
Baseline oxygen saturation, % Mean (± SD)	97.0 ± 1.30	97.2 ± 1.23	96.9 ± 1.36	96.7 ± 1.42	97.1 ± 1.24	0.025	0.012
Nadir oxygen saturation, Mean (± SD), %	94.1 ± 2.83	94.6 ± 2.38	93.5 ± 3.16	93.1 ± 3.31	94.0 ± 2.83	< 0.001	< 0.001
Oxygen saturation on recovery, Mean (± SD), %	96.6 ± 1.52	96.8 ± 1.37	96.4 ± 1.63	96.2 ± 1.66	96.6 ± 1.57	0.002	0.002
Chest X-ray abnormalities, n (%)							
No	271 (66.4)	174 (80.2)	97 (50.8)	52 (44.8)	45 (60.0)	< 0.001	< 0.001
Yes	137 (33.6)	43 (19.8)	94 (49.2)	64 (55.2)	30 (40.0)		
Consolidation	*17 (4.2)*	*2 (0.9)*	*15 (7.9)*	*10 (8.6)*	*5 (6.7)*	< *0.001*	*0.002*
GGO	*106 (26.0)*	*35 (16.1)*	*71 (37.2)*	*45 (38.8)*	*26 (34.7)*	< *0.001*	< *0.001*
Reticulation	*41 (10.1)*	*13 (6.0)*	*28 (14.7)*	*18 (18.1)*	*7 (9.3)*	*0.004*	*0.002*

^, 404 patients performed 6MWT: 217 had normal spirometry, 187 had abnormal spirometry (114 restrictive, 73 PRISm and obstructive); + , 402 patients performed 1MSTS: 215 had normal spirometry, 187 had abnormal spirometry (114 restrictive, 73 PRISm and obstructive); *, *p*-value for differences between patients with normal and abnormal spirometry; #, *p*-value for differences between patients with normal, restrictive pattern, and PRISm and obstructive pattern spirometry.

### Spirometry and factors associated with abnormal results

**Figure 2:**
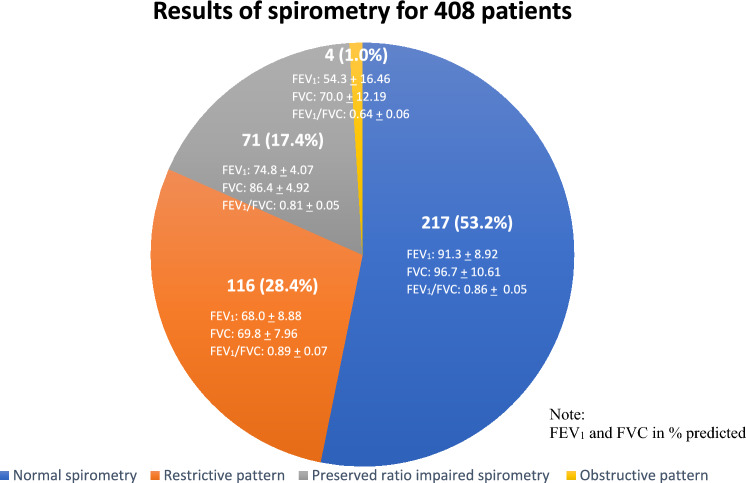
Results of spirometry for 408 patients.

Abnormal spirometry was detected in 46.8% of the patients, with 28.4% having a restrictive pattern, 17.4% having PRISm, and 1.0% having an obstructive pattern. The mean values of their FEV_1_, FVC, and FEV_1_/FVC are presented in Fig. 2.

Factors associated with abnormal spirometry results included patients’ age (*p* < 0.001), hypertension (*p* < 0.001), cardiovascular disease (*p* = 0.010), corticosteroids treatment (*p* = 0.006), IMV support (*p* = 0.042), ARDS (*p* = 0.035), pulmonary embolism (*p* = 0.007), ICU admission (*p* = 0.001), duration from discharge to follow-up (*p* = 0.001), oxygen desaturation with 6MWT (*p* = 0.002), oxygen desaturation with 1MSTS (*p* = 0.003), as well as the presence of consolidation (*p* < 0.001), GGO (*p* < 0.001), and parenchymal reticulation on chest X-ray (*p* = 0.004) (Table 4).

**Table 4:** Binary and multinomial logistic regression analyses to determine factors associated with abnormal spirometry results.

Parameters	Binary logistic regression*	Multinomial logistic regression^#^	
	Abnormal spirometry, OR (95% CI), *p*-value	Restrictive, OR (95% CI), *p*-value	PRISm and obstructive, OR (95% CI), *p*-value
Age^	1.0^a^ (1.01–1.04), 0.003	1.0 (0.98–1.03), 0.778	1.0^b^ (1.01–1.07), 0.004
Gender,			
Male	–	Ref	Ref
Female		2.1 (1.13–3.94), 0.019	0.5 (0.23–1.04), 0.062
Diabetes mellitus,			
No	–	Ref	Ref
Yes		0.8 (0.42–1.53), 0.501	0.6 (0.30–1.37), 0.249
Hypertension,			
No	Ref	Ref	Ref
Yes	1.5 (0.90–2.39), 0.123	1.8 (0.97–3.50), 0.061	1.1 (0.54–2.23), 0.800
Cardiovascular disease,			
No	Ref	Ref	Ref
Yes	3.5 (1.19–10.47), 0.023	3.1 (0.89–10.82), 0.075	3.1 (0.78–12.50), 0.108
Most severe illness during hospitalization,^!^			
Moderate	Ref	Ref	Ref
Severe	0.6 (0.27–1.28), 0.182	1.5 (0.37–6.50), 0.554	3.6 (0.79–16.23), 0.098
Critical	0.9 (0.41–2.22), 0.954	0.9 (0.42–2.08), 0.857	1.4 (0.47–3.98), 0.566
Received corticosteroids during hospitalization,			
No	Ref	Ref	Ref
Yes	1.3 (0.61–2.94), 0.475	1.3 (0.39–4.08), 0.703	1.5 (0.54–4.06), 0.439
Received hydroxychloroquine during hospitalization,			
No	–	Ref	Ref
Yes		0.5 (0.08–2.65), 0.388	4.4 (1.15–16.97), 0.030
Received immunomodulators during hospitalization,			
No	–	Ref	Ref
Yes		2.4 (1.07–5.28), 0.034	0.7 (0.23–2.36), 0.606
Required IMV,			
No	Ref	Ref	Ref
Yes	0.9 (0.44–1.95), 0.843	1.2 (0.53–2.85), 0.623	0.9 (0.29–3.11), 0.922
ARDS,			
No	Ref	–	–
Yes	–, < 0.001		
Pulmonary embolism,			
No	Ref	Ref	Ref
Yes	1.3 (0.73–2.22), 0.391	1.0 (0.53–2.00), 0.925	2.3 (1.07–5.13), 0.033
ICU admission,			
No	Ref	Ref	Ref
Yes	1.4 (0.80–2.46), 0.244	1.3 (0.62–2.62), 0.519	1.2 (0.51–2.85), 0.679
Length of hospital stay^	–	1.0 (0.99–1.04), 0.357	0.9^c^ (0.92–1.00). 0.099
Time from discharge to follow-up^	1.0 (1.00–1.02), 0.035	1.0 (0.99–1.00), 0.060	1.0 (0.99–1.00), 0.201
mMRC score^	–	1.2 (0.92–1.66), 0.164	0.7 (0.50–1.12), 0.158
6MWT distance^	–	1.0 (0.99–1.00), 0.501	1.0 (0.99–1.00), 0.678
6MWT oxygen desaturation,			
No	Ref	Ref	Ref
Yes	1.9 (1.20–3.06), 0.007	1.4 (0.76–2.52), 0.290	2.4 (1.20–4.75), 0.014
1MSTS oxygen desaturation,			
No	Ref	Ref	Ref
Yes	1.4 (0.85–2.30). 0.184	1.5 (0.81–2.79). 0.194	1.4 (0.70–2.91). 0.329
Consolidation on chest X-ray,			
No	Ref	Ref	Ref
Yes	8.1 (1.75–37.42). 0.008	10.1 (1.88–54.83), 0.007	8.6 (1.41–52.57), 0.020
GGO on chest X-ray,			
No	Ref	Ref	Ref
Yes	2.6 (1.52–4.30), < 0.001	2.2 (1.16–4.26), 0.016	2.6 (1.3–5.3), 0.009
Reticulation on chest X-ray,			
No	Ref	Ref	Ref
Yes	1.2 (0.55–2.68), 0.628	2.0 (0.81–4.81), 0.134	0.6 (0.19–1.87), 0.372

*, binary logistic regression analysis using patients with normal spirometry as reference; #, multinomial logistic regression analysis using patients with normal spirometry as reference; ^, continuous variables; !, Most severe illness during hospitalization was added as a covariate for binary logistic regression even though univariate *p* = 0.052; a, OR = 1.03; b, OR = 1.04; c, OR = 0.94.

Multivariate analyses using binary logistic regression showed that patients with underlying cardiovascular disease (OR 3.5, 95% CI 1.19–10.47, *p* = 0.023), those with oxygen desaturation during 6MWT (OR 1.9, 95% CI 1.20–3.06, *p* = 0.007), and those with consolidation (OR 8.1, 95% CI 1.75–37.42, *p* = 0.008) or GGO appearance (OR 2.6, 95% CI 1.52–4.30, *p* < 0.001) on chest X-ray were significantly more likely to have abnormal spirometry.

### Restrictive pattern, obstructive pattern, and PRISm

Multinomial logistic regression, using normal spirometry as a reference, showed that female patients (OR 2.1, 95% CI 1.13 -3.94, *p* = 0.019), those treated with immunomodulators (OR 2.4, 95% CI 1.07–5.28, *p* = 0.034), those with consolidation (OR 10.1, 95% CI 1.88–54.83, *p* = 0.007) or GGO appearance (OR 2.2, 95% CI 1.16–4.26, *p* = 0.016) on chest X-ray were significantly more likely to show a restrictive pattern spirometry. Conversely, patients treated with hydroxychloroquine (OR 4.4, 95% CI 1.15–16.97, *p* = 0.030), those who developed pulmonary embolism (OR 2.3, 95% CI 1.07–5.13, *p* = 0.033), those with oxygen desaturation in the 6MWT (OR 2.4, 95% CI 1.20–4.75, *p* = 0.014), those with consolidation (OR 8.6, 95% CI 1.41–52.57, *p* = 0.020) or GGO appearance (OR 2.6, 95% CI 1.3–5.3, *p* = 0.009) on chest X-ray were significantly more likely to show PRISm and obstructive pattern spirometry.

### Findings on body plethysmography, diffusion capacity, and HRCT of the lungs

Eighty-nine patients underwent body plethysmography and diffusion capacity assessment, revealing a mean RV of 57.8 ± 39.08% predicted, a mean TLC of 65.1 ± 13.25% predicted, a mean DLCO of 62.5 ± 13.94% predicted, and a mean DLCO/Va of 103.6 ± 17.52 (Table 5). Of 80 patients who underwent HRCT of the lungs, 81.3% had GGO, 52.5% had OP, 85.0% had parenchymal reticulation, and 33.8% showed other findings. The mean CT score for these patients was 9.8 ± 5.96.

**Table 5:** Findings of body plethysmography, diffusion capacity, and HRCT of the lungs for patients with restrictive pattern spirometry.

Parameters of body plethysmography, and diffusion capacity	Total number of patients, n = 89	Findings of lungs HRCT	Total number of patients, n = 80
FEV_1_, Mean (± SD), % predicted	76.8 ± 13.97	HRCT abnormalities, n (%)	
FVC, Mean (± SD), % predicted	75.6 ± 14.00	No	6 (7.5)
FEV_1_/FVC, Mean (± SD), %	0.86 ± 0.12	Yes	74 (92.5)
RV, Mean (± SD), % predicted	57.8 ± 39.08	GGO	
TLC, Mean (± SD), % predicted	65.1 ± 13.25	OP	*65 (81.3)*
DLCO, Mean (± SD), % predicted	62.5 ± 13.94	Reticulation	*42 (52.5)*
DLCO/Va Mean (± SD), % predicted	103.6 ± 17.52	Others	*68 (85.0)*
			*27 (33.8)*
		CT-scores, Mean (± SD), score	9.8 ± 5.96

## Discussion

The current study highlights that nearly half of the patients hospitalized for moderate-to-critical COVID-19 continue to show abnormal spirometry even after an average of five months after discharge. Approximately one-third of them displayed a restrictive pattern, while another one-fifth surprisingly manifested PRISm. This study identifies chest X-ray as a reliable tool for predicting abnormal spirometry and its subtypes, particularly when consolidation and GGO are present. Furthermore, the 6MWT could be a valuable tool for predicting abnormal spirometry. Although certain clinical data were also found to be useful, the 1MSTS and PROs do not add additional value to the prediction of spirometry abnormalities.

A meta-analysis of seven studies, primarily conducted in China, revealed that 22.9% of patients hospitalized for COVID-19 demonstrated abnormal spirometry within three months post-discharge^34^. Among these, 15.0% exhibited a restrictive pattern while 7.9% showed an obstructive pattern^34^. A separate study in Thailand reported abnormal spirometry in 17.2% of patients hospitalized for mild-to-severe COVID-19 at sixty days post-discharge, with 9.2% having an obstructive pattern and 8.0% having a restrictive pattern^35^. In Spain and Belgium, studies reported solely a restrictive pattern among hospitalized COVID-19 patients. In the Spanish study, 14.3% of patients requiring oxygen supplementation for pneumonia exhibited this pattern at two months, 9.3% at six months, and 6.7% at twelve months^36^. In the Belgian study, 55% of patients admitted to the ICU for ARDS demonstrated a restrictive pattern at three months^37^. Compared to these other studies, our study showed a high prevalence of abnormal spirometry potentially attributed to the predominance of severe and critical COVID-19 cases among our cohort. The observation that the restrictive pattern was the most common spirometry abnormality aligns with findings in China^34^, France^38^, Spain^36^, and Belgium^37^. The increased proportion of patients with an obstructive pattern in the Thailand study, however, could be due to the non-exclusion of individuals with pre-existing lung diseases, including bronchial asthma and chronic obstructive pulmonary disease (COPD)^35^.

The majority of existing studies have focused on investigating the lung function of patients recovering from COVID-19 based on severity of illness. These studies have consistently shown that individuals with more severe illness tend to exhibit significantly lower static lung volumes and diffusion capacity, while their dynamic lung volumes in spirometry often remain preserved^8,38–41^. To date, only a study in Thailand and China have respectively reported significantly lower dynamic lung volumes in patients with more severe illness^35,42^, while another study in the Netherlands found only FVC to be significantly lower in such cases^43^. Additional studies have shown that for individuals post-COVID-19, spirometry indices were not significantly different from the healthy population^44^, those with other viral upper respiratory tract infections^45^, or the same cohort of patients one year before the infection^46^. A review by Thomas et al. further concluded that spirometry indices are often well-preserved in COVID-19, without being significantly affected by illness severity^47^. As far as we know, our study is the first to demonstrate no significant differences in spirometry patterns between patients with varying severity of COVID-19.

Our study identifies several factors associated with abnormal spirometry in patients recovering from COVID-19, notably abnormal chest X-ray and 6MWT during follow-up, as well as underlying cardiovascular disease. In Thailand, individuals with abnormal chest X-ray after COVID-19 had significantly lower dynamic lung volumes^35^. Additionally, chest CT abnormalities after COVID-19 were correlated with lower dynamic lung volumes and diffusion capacity among those in Austria^48^, Netherlands^40^, and China^49^, although not in France^38^. Oxygen desaturation during the 6MWT was associated with diffusion capacity impairment among post-COVID patients in the Netherlands^40^, but not in Thailand or Germany^35,50^. The relationship between lung function and mMRC scores or 6MWT in individuals post-COVID has not been extensively explored in previous studies, where these measures were assessed but not specifically analyzed for their association or correlation^36,41,51,52^. To date, only one study from Austria has reported a negative correlation between lung function and mMRC scores^48^, while another study from the Netherlands reported an association between poor lung function and oxygen desaturation in the 6MWT^40^. Additionally, two other studies, one from China and another from Belgium, found concurrent abnormalities in lung function, radio-imaging, 6MWT, and mMRC in the same cohort of post-COVID patients, suggesting a potential relationship between these factors^8,37^. Other factors associated with impaired lung function in previous studies included older age^36,46^, female gender^36^, lower body mass index^36^, underlying chronic lung disease^46^, higher inflammatory markers at presentation^36,49^, previous ARDS^37^, and shorter discharge-to-follow-up interval^48,51^. Corticosteroid treatment was linked to better lung function recovery^37,51^, while this was not observed with other treatment modalities^43^. Overall, our study findings are consistent with most of other studies.

Our study is the first to report PRISm in post-COVID patients. PRISm, previously known as pre-COPD, restrictive, or non-specific pattern, has a prevalence of 4.7–22.3% in the general population^53^. Recent studies indicate that it primarily affects the small airways and vessels while sparing lung parenchyma^53,54^. Two studies have shown that 25.1% and 32.6% of individuals with PRISm, respectively progress to spirometry-defined COPD in five years^55,56^. Conversely, improvement of spirometry from obstructive pattern to PRISm over time has also been observed^57^. Therefore, individuals with PRISm in this study could either indicate an improvement from airflow obstruction or an early sign of deterioration to COPD after COVID-19. Additionally, the possibility that this reflects population prevalence rather than being directly attributed to COVID-19 cannot be discounted. Future studies that prospectively following up on this patient cohort could provide a definitive answer. The high prevalence of the restrictive pattern among our patients can be explained by the aberrant wound healing typically following diffuse alveolar damage by SARS-CoV-2, leading to severe scarring and fibrosis^58^. Respiratory muscle weakness following SARS-CoV-2 infection could also be another possibility^59^.

The findings from this study have several clinical implications. First, spirometry should be routinely performed in patients post moderate-to-critical COVID-19 due to the high prevalence of abnormality. Second, when universal spirometry screening is not feasible among them, a targeted risk stratification approach considering chest X-ray, 6MWT, and specific clinical characteristics is recommended. Third, chest X-ray proves to be the most reliable screening tool for abnormal spirometry, with the additional benefits of being readily available and cost-effective. Treating clinicians and radiologists should focus on detecting consolidation and GGO features. Fourth, the 6MWT also emerges as a valuable screening tool for abnormal spirometry. Fifth, 1MSTS and PROs may not add significant value to the screening and should not be prioritized during follow-up. Sixth, this study suggests the potential development of a scoring system that combines these factors, providing a practical tool for clinicians to efficiently select patients for lung function tests.

The large sample size of this study allows for the generalizability of the result. It is one of the few studies in the Southeast Asia, where outcomes may differ from other parts of the world due to variations in genetic, environmental, and lifestyle factors. The study focused on patients hospitalized with moderate-to-critical COVID-19 who were more susceptible to long-term lung injuries. The comprehensive study outcomes include objective assessments like lung function, radio-imaging, and cardiopulmonary functional evaluations, alongside subjective assessments such as PROs. However, this study was conducted during the peak of the pandemic. Travel restrictions, public reluctance to visit hospitals, and constrained healthcare resources could lead to several weaknesses. First, the convenience sampling method may introduce bias. Second, not every patient can undergo examination with body plethysmography, for diffusion capacity, and with HRCT. Third, some patients who were offered these investigations defaulted. Fourth, due to practical and logistic reasons the follow-up assessments could not be conducted at a fixed interval, such as three months, six months, or twelve months post-discharge. Fifth, no spirometry was done before the COVID-19 infection to demonstrate baseline normality. Sixth, factors associated with specific abnormal spirometry patterns should be interpreted with caution, as the sample size may not be powerful enough to accurately reflect these secondary outcomes. Seventh, although patients’ age and discharge-to-follow-up interval may show statistical significance in multivariate analysis, an OR of 1.0 indicates a lack of clinical significance. Eighth, the OR for ARDS could not be generated using logistic regression due to the complete separation phenomenon and the events were extremely low resulting in a lack of statistical power for the analysis. Ninth, multidimensional assessment of PROs such as HRQOL was not performed. Lastly, lung function tests were not conducted as a follow-up after the study to observe potential changes in patterns.

## Conclusions

Patients recovering from moderate-to-critical COVID-19 often demonstrated abnormal spirometry, particularly manifesting a restrictive pattern and PRISm. Therefore, spirometry should be routinely offered to those at higher risk of abnormalities, such as individuals with abnormal chest X-ray and 6MWT during follow-up, as well as those with underlying cardiovascular disease. PRISm represents a novel finding among post-COVID patients, warranting further follow-up to elucidate the underlying mechanism of this lung function abnormality.

## Data Availability

The datasets used and/or analysed during the current study are available from the corresponding author on reasonable request.
